# Sphingomyelin Synthase 2 Participate in the Regulation of Sperm Motility and Apoptosis

**DOI:** 10.3390/molecules25184231

**Published:** 2020-09-15

**Authors:** Xiatian Li, Tao Luo, Hua Li, Nianlong Yan

**Affiliations:** 1 Department of Biochemistry and Molecular Biology, Faculty of Basic Medical Science, Nanchang University, Nanchang 330031, China; summer5388@163.com (X.L.); lihua880303@163.com (H.L.); 2 Institute of Life Science, Nanchang University, Nanchang 330031, China; luotao.ncu@hotmail.com

**Keywords:** SMS2, Akt, ERK, sperm motility

## Abstract

Sphingomylin participates in sperm function in animals, and also regulates the Akt and ERK signaling pathways, both of which are associated with the asthenospermia. Sphingomyelin synthase 2 (SMS2) is involved in the biosynthesis of sphingomylin. To determine the relationship between SMS2 and human sperm function, we analyzed the distribution of SMS2 in human sperm and testes, and SMS2 expression in patients with asthenospermia and normozoospermia; human sperm were treated with anti-SMS2, and the sperm motility, penetration ability into methylcellulose, capacitation and acrosome reaction, and sperm [Ca^2+^]i imaging were evaluated, while the Akt and ERK pathway and cleaved caspase 3 were also analyzed. Results showed that SMS2 was localized in the testis and human sperm, and the protein levels of normozoospermia were higher than asthenospermia. Inhibition of SMS2 activity significantly decreased sperm motility and penetration ability into methylcellulose, but had no influence on capacitation and acrosome reaction, or on intracellular [Ca^2+^]i compared to IgG-treated control groups. Moreover, the phosphorylation level of Akt was decreased, whereas the phosphorylation of ERK and cleaved-caspase 3 levels were significantly increased. Taken together, SMS2 can affect sperm motility and penetration ability into methylcellulose, and participate in apoptosis associated with the Akt and ERK signaling pathways.

## 1. Introduction

Infertility is a common reproductive disease, and male infertility accounts for half of the cases [1]. A significant proportion of male infertility cases is accompanied by abnormal semen quality including asthenospermia, teratospermia, oligozoospermia, and azoospermia [1]. However, asthenospermia, which is associated with poor sperm viability and motility, is the leading factor in male infertility [1,2]. Many causes of asthenospermia have been reported so far [3,4,5], which include dysplasia of fibrous sheath, defects in the action of cilium, disfunction of energy metabolism, dysplasia of calcium channel [3,4]. Insufficiency of essential fatty acid, such as DHA, is also a cause of this disease [5]. Moreover, signaling pathways are also associated with asthenospermia [6]. Akt is a serine/threonine protein kinase that plays a critical role in controlling cell survival and apoptosis; it also participates in spermatogenesis and sperm motility [7]. For example, Kim et al. found that apoptotic sperm cells were more prevalent in null mice (both Akt1^−/−^ and Akt2^−/−^ male mice) than in wild-type mice, whereas sperm concentration and motility were significantly lower in null mice [8]. Moreover, some reports indicated that the viability and motility of sperm cells are tightly related with the activation of the Akt signaling pathway, whereas oxidative stress, drugs, and ischemic injury are the factors that can inhibit the Akt signal pathway by blocking its phosphorylation, leading to reduced viability and motility of the sperm [9,10,11,12]. In addition to the Akt signaling pathway, the ERK signaling pathway is a key regulatory molecule participating in diverse cellular functions such as growth, differentiation, and apoptosis. In terms of male reproduction, the ERK signaling pathway can modulate the sperm maturation in the initial segment of the epididymis, and affect the capacitation, acrosome reaction, and motility [13,14,15]. For example, Seong et al. discovered that activation of ERK can induce the mitochondria-dependent apoptosis pathway of sperm induced by CdCl_2_, which can decrease the sperm motility of mice [15]. Thus, both the Akt and ERK signaling pathways can regulate sperm motility.

Sphingomyelin (SM) is a dominant sphingolipid in the membranes of mammalian cells. Sphingomyelin synthase (SMS), the key enzyme participating in the biosynthesis of SM, has two isoforms, SMS1 and SMS2, which are found on the Golgi apparatus and Golgi apparatus and plasma membranes, respectively [16]. Some studies demonstrated that SM was involved in the spermatogenesis and the functions of animal sperm cells [17,18,19]. In 1992, Robinson et al. identified a novel species of SM in rat testis and boar spermatozoa [17]. Thereafter, Zanetti et al. confirmed that SM existed in rat and mouse testis and rat spermatozoa, and the teleological objective of fulfilling their ultimate physiological role in spermatozoa is to fertilize an oocyte [18]. In detail, SM can influence the rate of capacitation by slowing the loss of sterols [19]. Moreover, Lee et al. and Wittmann et al. suggested that SMS may play a crucial role in sloughing of spermatocytes and spermatids, causing progressive infertility of male homozygotes, acrosome formation, and plasma membrane restructuring from late round spermatids to early elongating spermatids by affecting the lipid metabolism in mice [20,21]. Clearly, SMS are associated with male infertility.

As mentioned above, SMS and the Akt and ERK signaling pathways are all associated with male infertility, but their connection is not clear in sperm motility. Thus, in this study, we aimed to identify the role of SMS2 in asthenospermia and the relationship between SMS2 and the Akt and ERK signal pathways in human sperm viability and motility.

## 2. Results

### 2.1. SMS2 Can be Expressed in the Human Testis and Present in Whole Sperm

**Figure 1:**
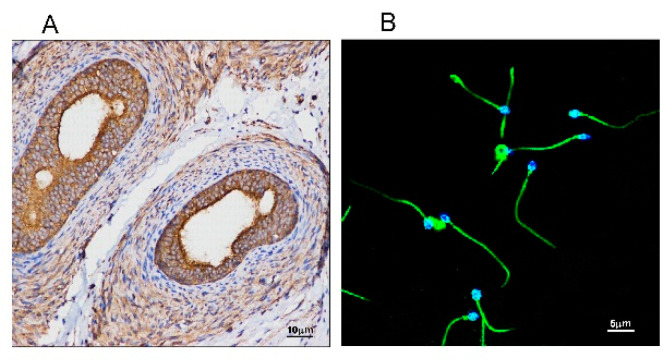
The distribution of sphingomyelin synthase 2 (SMS2) in testes and sperm. (**A**) Analysis of SMS2 protein levels in a human testis by immunohistochemistry; (**B**) analysis of SMS2 in human sperm by immunofluorescence.

Since SM is the main component of cellular membranes and SMS2 is the major enzyme catalyzing SM biosynthesis, we measured the expression of SMS2 in testis and sperm cells by immunohistochemistry and immunofluorescence, respectively. As shown in Figure 1A, SMS2 was expressed in the testis, with higher expression in the efferent ductules. Thereafter, we investigated the expression of SMS2 in the human sperm cells by immunofluorescence. As shown in Figure 1B, SMS2 was localized not only in the head and tail, but on the whole sperm cells; however, fluorescence intensity on the tail was stronger than that on the head in mature human spermatozoa.

### 2.2. The Expression of SMS2 Was Different in Asthenospermia and Normozoospermia

**Figure 2:**
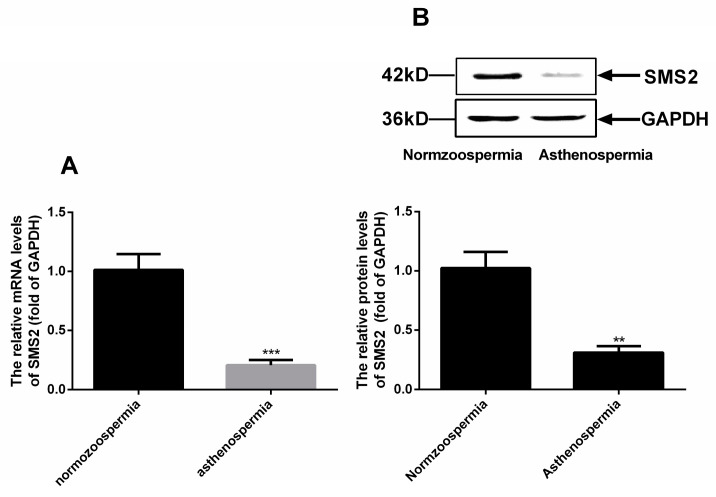
The expression of SMS2 in asthenospermia and normozoospermia. (**A**) mRNA levels of SMS2, *** *p* < 0.001; (**B**) protein levels of SMS2. Values shown are the mean ± SD (*n* = 20), ** *p* < 0.005 vs. normozoospermia.

Based on the above results, we continuously measured the mRNA and protein level of SMS2 in normozoospermia and asthenospermia by reverse transcription-quantitative polymerase chain reaction and Western blot analysis. The results (Figure 2A) showed that the SMS2 mRNA levels were obviously decreased by about 80.67 + 4.38% (*p* < 0.001, *n* = 20), and protein levels were significantly reduced to approximately 71.33 ± 8.47% (*p* < 0.005, *n* = 20) in asthenospermia compared to normozoospermia (Figure 2B).

### 2.3. The SMS2 Antibody Can Block the SMS Enzyme Activity and Biosynthesis of SM

**Figure 3:**
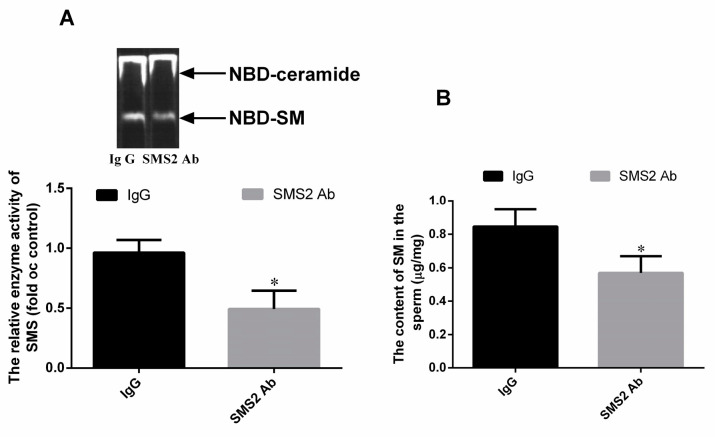
The enzyme activity of SMS and SM content. (**A**) The enzyme activity of SMS; (**B**) the SM content in sperm. Values shown are the mean ± SD (*n* = 3), * *p* < 0.05 vs. IgG group.

To verify whether the SMS2 antibody can block the SMS enzyme, this study detected the enzyme activity of SMS by thin layer chromatography (TLC) assay. From Figure 3A, we found that 20 μg/mL of SMS2 antibody (1:50) can inhibit the SMS enzyme activity compared with the IgG group, the enzyme activity of SMS decreased about 47.0 ± 5.32% (* *p* < 0.05, *n* = 3). Thereafter, we also measured the SM content of the sperm. From Figure 3B, we found that the SM content in the SMS2 Ab group decreased about 27.67 ± 4.85% compared to the IgG group.

### 2.4. Inhibition of SMS2 Activity Decreased Sperm Motility and Penetration Ability into Methylcellulose 

**Figure 4:**
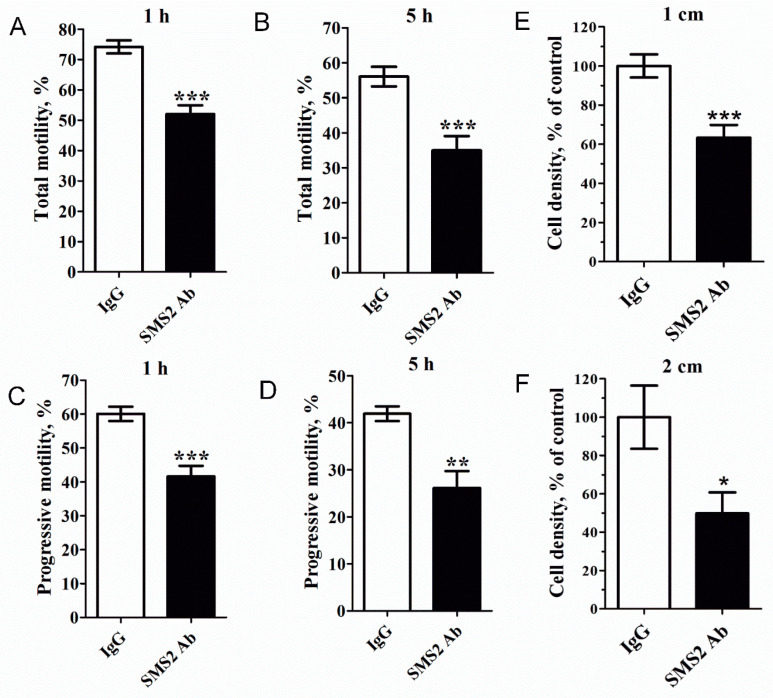
The effect of SMS2 inhibition on sperm motility and penetration ability into methylcellulose. (**A**–**D**) Inhibition of the SMS2 activity affects sperm motility; **(E**, **F)** the effect of SMS2 inhibition on penetration ability into methylcellulose. Values shown are the mean ± SD (*n* = 9). * *p* < 0.05, ** *p* < 0.005, and *** *p* < 0.001 vs. IgG group.

Next, sperm cells were treated with anti-SMS2 and the CASA (Computer-aided sperm analysis) system was used to analyze the motility of human spermatozoa. As shown in Figure 4A–D, inhibition of SMS2 activity could significantly reduce the total and progressive motility of sperm cells (* *p* < 0.05 and *** *p* < 0.001, respectively, *n* = 9). Compared with the IgG control groups, after 1 h, the total motility decreased from 71.53 ± 3.02% to 51.42 ± 4.22% (Figure 4A), while progressive motility decreased from 59.50 ± 2.40% to 42.84 ± 3.67% (Figure 4C), but after 5 h, the total and progressive sperm cell motility decreased from 50.39 ± 5.24% to 30.50 ± 5.25% (Figure 4B) and from 36.94 ± 4.89% to 22.5 ± 4.35% (Figure 4D), respectively. Obviously, the inhibition of SMS2 disturbed the total and progressive motility of spermatozoa, and this effect was proportional to the duration of inhibition, that is, the longer the treatment with SMS2 antibody, the lower the total and progressive motilities of sperm cells. It was expected that the sperm cell motility should be reflected in its penetration ability into viscous medium. Therefore, the penetration of anti-SMS2-treated sperm cells into methylcellulose was assessed. The results (Figure 4E,F) showed that the anti-SMS2 treatment could also inhibit the sperm penetration ability into methylcellulose. Compared to the IgG groups, the penetration number of the anti-SMS2 treated cells was significantly different at 1 cm (Figure 4E, * *p* < 0.001, *n* = 9) and at 2 cm (Figure 4F, * *p* < 0.05, *n* = 9), and the cell density decreased by about 36.62% and 50.17%, respectively. Altogether, these results verified that augmentation of the SMS2 activity increased both the sperm motility and the penetration ability into methylcellulose, however, inhibition of the SMS2 activity could reverse this response.

### 2.5. Inhibition of SMS2 Activity Had no Significant Effect on the Capacitation and Acrosome Reaction

**Figure 5:**
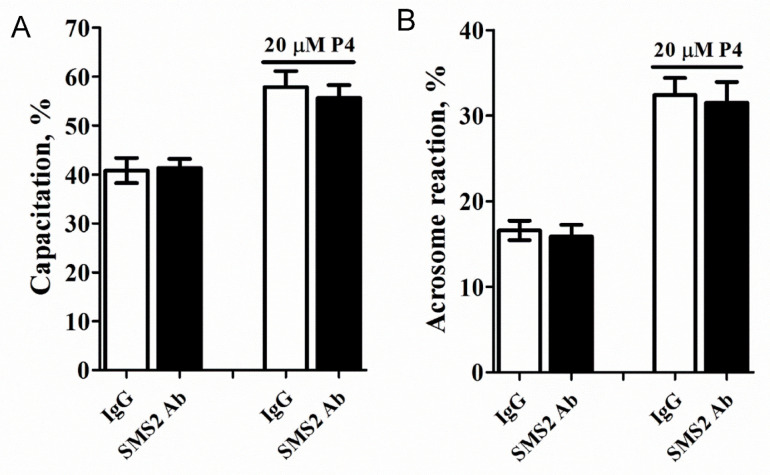
The effect of inhibition of the SMS2 activity on the capacitation and acrosome reaction. (**A**) The effect of inhibition of the SMS2 activity on capacitation. (**B**) The effect of inhibition of the SMS2 activity on the acrosome reaction.

Capacitation and acrosome reaction of mammalian spermatozoa are essential for oocyte penetration, thus, we tested whether these functions were affected in the anti-SMS2-treated sperm cells. These results (Figure 5A,B) demonstrated that inhibition of the SMS2 activity and addition of 20 µM progesterone slightly reduced the capacitation and acrosome reaction, respectively, (Figure 5A,B, *p* > 0.05, *n* = 9) but without any statistical significance.

### 2.6. Inhibition of SMS2 Activity had no Effect on Sperm Ca^2+^ Signaling

**Figure 6:**
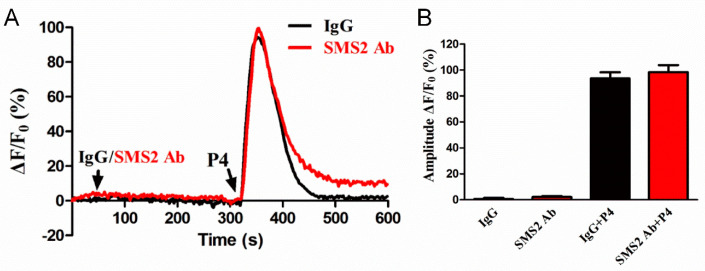
The effect of SMS2 inhibition on sperm Ca^2+^ signaling. (**A**) The effects of SMS2 inhibition on the time of maximum intracellular [Ca^2+^]i; (**B**) the effects of SMS2 inhibition on the ratio of ΔF/F0.

Sperm motility and penetration may depend on the intracellular Ca^2+^ concentration ([Ca^2+^]i); therefore, we sought to investigate this. The sperm were first incubated with IgG and SMS2 antibody for 280 s and then progesterone was added for 280 s. The realtime curves showed that both IgG and SMS2 antibody did not change the sperm [Ca^2+^]i (40–320 s, Figure 6A) and progesterone-induced [Ca^2+^]i (360 s, Figure 6A). The statistical analysis showed that the amplitudes of ΔF/F0 that indicate the changes of sperm [Ca^2+^]i were not significantly different between IgG and SMS2 antibody at the times of 80 s and 360 s (Figure 6B, *p* > 0.05, *n* = 9). In conclusion, SMS2 could not affect the [Ca^2+^]i, and that other factors may modulate the sperm motility and penetration ability into methylcellulose.

### 2.7. Inhibition of SMS2 Activity Affects the Phosphorylation of AKT and ERK as well as the Cleaved Caspase 3

**Figure 7:**
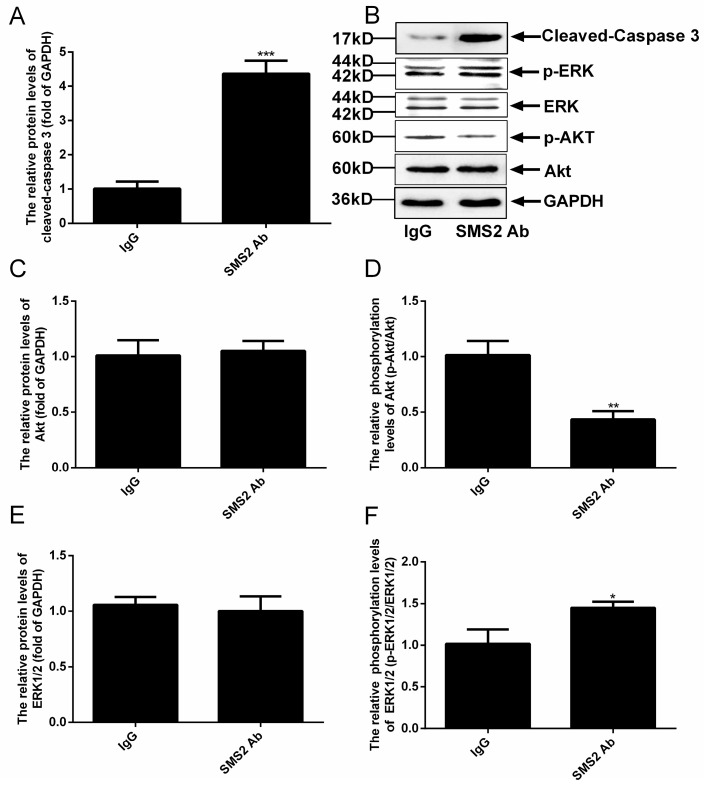
Analysis the ERK and Akt signal pathways and cleaved-caspase 3 by Western blot. (**A**) The relative protein levels of cleaved-caspase 3; (**B**) the results of Western blot of cleaved-caspase 3, Akt, ERK and GAPDH; (**C**) the relative protein levels of Akt; (**D**) the phosphorylation levels of Akt; (**E**) the relative protein levels of ERK; (**F**) the phosphorylation levels of ERK. Values shown are mean ± SD (*n* = 5). * *p* < 0.05, ** *p* < 0.005, and *** *p* < 0.001 vs. IgG group.

Previous studies reported that the Akt and ERK pathways could affect the sperm cell vitality and motility [9,10,11,12,13,14]. When we measured the phosphorylation levels of Akt and ERK, our results revealed that the inhibition SMS2 activity could significantly reduce the level of Akt phosphorylation by approximately 57.83 ± 8.59%, (Figure 7D, *p* < 0.005, *n* = 5), but had no effect on the protein level of Akt (Figure 7C). In contrast, when the SMS2 activity was inhibited, the phosphorylation level of ERK was increased significantly, by 43.00 ± 10.80% (Figure 7F, *p* < 0.05, *n* = 5), without, however, affecting the protein level of ERK (Figure 7E). Both Akt and ERK can regulate the processes of proliferation, differentiation, and apoptosis; therefore, the alteration of Akt and ERK phosphorylation could lead to changes of the sperm vitality and could even induce sperm apoptosis. The results presented in Figure 7A show that inhibition of SMS2 activity significantly affected the activity of caspase 3 (the cleaved caspase 3) (*p* < 0.001, *n* = 5), which increased by 335.00 ± 25.46 %.

## 3. Discussion

Many factors can affect the asthenospermia including fibrous sheath, cilium, energy metabolism, calcium channel and essential fatty acids [3,4,5]. However, in this study, we found that SMS2 was related with asthenospermia. Our initial search for the expressional distribution of SMS2 in the human testis revealed that SMS2 was indeed expressed in the testis and in particular in the efferent ductules, where it can affect the development of spermatogenic cells. Thus, our results suggested that SMS2 may play some role(s) in spermatogenesis. In fact, Wittmann et al. also showed similar results for SMS1 in mice, wherein SMS1 plays a wider role in testis polyunsaturated fatty acid homeostasis and in male fertility [21]. Moreover, when we tested the SMS2 expression in sperm cells, we were able to show that the SMS2 was located mainly on the head and tail of sperm cells. In agreement with this, SMS catalyzes the biosynthesis of SM which is found on both the head and tail of rat spermatozoa [22]. In somatic cells, SMS2 is located in the Golgi apparatus and plasma membranes [16]; however, sperm cells do not contain Golgi apparatus, which is deformed into the acrosome and head of the sperm cell [23]. The distribution of SMS2 was found throughout the whole human sperm cell, which is of course, indicative that SMS2 may be involved in human spermatozoa. As support for the potential role of SMS2 in spermatogenesis, we confirmed that SMS2 may be related with sperm function after estimating SMS2 expression in asthenozoospermia and normozoospermia, especially the sperm motility, since the expression of SMS2 was decreased in asthenospermia compared to the normozoospermia.

In early 2007, Lee et al. [20] found that SMS2 may play a crucial role in the lipid metabolism in acrosome formation and plasma membrane restructuring of late round spermatids to early elongating spermatids in rats. This is consistent with our SMS localization experiment results that SMS2 was distributed at the head and tail of the sperm cell, which are mainly related with the acrosome reaction and motility, respectively [24]. Nevertheless, through the inhibition of SMS2 activity in sperm cells we were able to show that SMS2 does not regulate the capacitation and acrosome reaction, consistent with the lack of effect on the intracellular [Ca^2+^]i levels. Interestingly, when we measured the motility of human sperm cells, we were able to show that the sperm motility and penetration ability into methylcellulose could be affected by SMS2, and the longer the SMS2 antibody-treatment lasted, the poorer the sperm motility outcome was. Many factors could be involved in the regulation of sperm motility and penetration ability into methylcellulose, including alteration of the intracellular [Ca^2+^]i levels. However, as we indicated above, we were not able to observe any such change. Clearly, these results suggested that other factors may affect sperm motility and penetration ability into methylcellulose.

Several studies have verified that the ERK signaling pathway participates in the male reproductive system functions, such as spermatogenesis and sperm motility [13,14,15,25]. Activation of ERK1/2 would block the function of sperm cells and increase their rate of apoptosis [26,27]. Hence, we further investigated the ERK signaling pathway and provided evidence that ERK1/2 phosphorylation levels were increased in the SMS2 antibody-treated human sperm cells without altering the expression of total ERK1/2. This is in agreement with the study by Ali A Shati, who showed that cisplatin could induce testicular damage and reproductive dysfunction in rats, which lowered the sperm cell count and motility and increased the morphological abnormalities of sperm cells by increasing the phosphorylation of ERK1/2 [10]. Moreover, it was shown that the addition of an ERK substrate peptide in demembranated fowl sperm could significantly decrease the MAPK/ERK signaling pathway and sperm motility [28]. Obviously, activation of the ERK1/2 signaling pathway is negatively correlated with sperm motility. Ali A Shati also discovered that cisplatin inhibited the Akt signaling pathway and induced apoptosis by increasing the levels of cleaved-caspase 3, which indicated that reduced sperm count and motility may also be related with the Akt signal pathway. In this study, we obtained similar results when human sperm cells were treated with an anti-SMS2 antibody by estimating the Akt/ERK pathway activity. Obviously, the results from our study and that of Ali A Shati demonstrated that both the activation of the ERK and the inhibition of the Akt signaling pathways could lower sperm motility. Bhaswati Banerjee et al. also reported similar results: when they treated the germ cell population from adult male Wistar rats with Benzo(a)pyrene, the sperm cell count and motility were decreased. Mechanistically, increased apoptosis of sperm cells correlated on one hand with activation of the ERK signaling pathway, and on the other hand with inhibition of the Akt signaling pathway [29]. Together, these results suggested that SMS2 regulated the sperm motility and penetration ability into methylcellulose, which may be related with the induction of apoptosis.

SMS2 catalyzes the conversion of ceramide and phosphatidylcholine (PC) to SM and diacylglycerol (DAG) [15]. Both ceramide and DAG are second messengers, which can regulate cell apoptosis and proliferation, respectively [30]. When the inhibition of SMS2 activity was blocked, DAG and PC levels would decrease, but SM and ceramide levels would increase. In fact, in our study, we did find that the SM content in the SMS2 Ab group was decreased compared to the IgG group, which suggested that the ceramide also could be decreased. Ceramide, as a second messenger, participates in many signaling pathways, including the ERK and Akt pathways [31,32]. For example, Jiang et al. found that C2-ceramide inhibited cell growth and proliferation via the Akt and ERK signaling pathways in cancer cells, and the angiogenesis too [31,32]. Meanwhile, Suomalainen et al. also provided evidence that ceramide could induce apoptosis of the sperm cells: their study found that during apoptosis, the ceramide levels increased rapidly before the appearance of activated caspase 3, which suggested a role for ceramide in the induction of germ cell death [33]. Moreover, the inhibition of SMS2 activity also can affect the transmembrane signal transduction via the lipid raft. The lipid raft is a microdomain located at the membrane; both SM and cholesterol are the main components of it [34]. Some studies reported that both the Akt and ERK signaling pathways can be regulated by the lipid raft [35,36]. For example, lipid raft reorganization led to Akt dephosphorylation in mantle cell lymphoma cells, which can inhibit the cells’ survival and contribute to chemotherapy [35]. Moreover, Zhang et al. suggested that lipid rafts provide a platform to inhibit the EGFR regulation of MMP-1 in SiHa cells through the MAPK/ERK signaling pathway [36]. Therefore, the inhibition of SMS2 activity may affect the SM content in lipid rafts and, thus, inhibit transmembrane signal transduction of the Akt signaling pathway in sperm cells [37].

**Figure 8:**
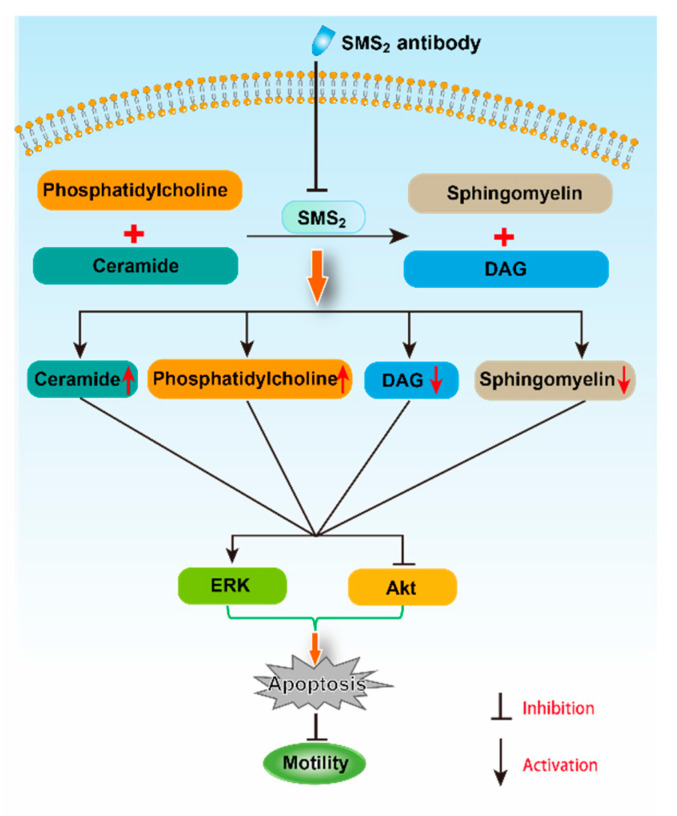
The possible mechanism for the effect of SMS2 on sperm motility.

Altogether, the inhibition of SMS2 activity could reduce the Akt phosphorylation and increase the ERK phosphorylation, which would increase human sperm apoptosis, and finally, would decrease both the sperm motility and penetration ability into methylcellulose (Figure 8). However, further study is necessary to uncover the detailed mechanism with which SMS2 regulates the Akt and ERK pathway.

## 4. Materials and Methods

### 4.1. Sample Collection and Treatment

Semen samples including 20 asthenospermia and normozoospermia, were collected from Jiang Xi Maternal and Child Health Hospital. Semen donors were of proven fertility and met the standard criteria for normozoospermia which refers to sperm count >40 million/mL and motility >50% [38]. On the contrary, samples with counts <40 million/mL and motility ≤32% are classified as asthenospermia. The three human testes were provided by Dr Tao Zeng from the Second Affiliated Hospital of Nanchang University. The Institutional Ethics Committee (NDSYDWLL-2018213) on human subjects had approved the collection of these samples.

### 4.2. Analysis of SMS2 in Human Testis and Sperm

Immunohistochemistry was used to measure the expression and distribution of SMS2 in the human testes. In brief, the human testes were fixed with 4% formaldehyde, embedded in paraffin, and cut into 4-μm-thick sections [39]. Next, sections were incubated in methanol with 0.3% H_2_O_2_, and boiled in 0.01 M sodium citrate (pH 6.0) for 10 min. Finally, the sections were incubated overnight with anti-SMS2 antibody (Abcepta, cat. no. AP9740b), and restained with hematoxylin. The distribution of SMS2 in the human sperm cells was analyzed by immunofluorescence. First, the sperm cells were washed and smeared on polylysine slides, and then allowed to air-dry. Next, the sperm cells were permeabilized with Triton X-100 (0.5%) after being washed with PBS. Subsequently, the sperm cells were treated with 3% BSA to block nonspecific binding. At the same time, the sperm were treated with polyclonal anti-SMS2 (1:50) antibody, washed three times by PBS, and then incubated with a secondary antirabbit (Kangwei, Beijing, China). Finally, slides were viewed with an inverted fluorescence microscope (IX-71, Olympus, Shinjuku, Tokyo, Japan) [40].

### 4.3. SMS Enzyme Activity Assay

The SMS activity of sperm was measured as previously described [37,41]. Briefly, sperm were homogenized in a buffer containing 50mM Tris-HCl, 1mM EDTA, and 5% sucrose. The homogenate was centrifuged at 12,000 rpm at 4 °C for 10 min, and the supernatant was used to analyze SMS activity. The supernatant was divided into two groups, IgG and SMS2 Ab. Both the IgG and SMS2 Ab groups were treated with 20 μg/mL rabbit secondary antibody and SMS2 antibody, respectively, in the reaction system (695 μL) contained 50 mM Tris-HCl (pH 7.4), 25 mM KCl for 30 min. Thereafter, 2 μL C6-NBD-ceramide (0.1 μg/mL; cat. no. 62527; Cayman chemical company, Ann Arbor, MI, USA) and 3 μL phosphatidylcholine (0.01 mg/mL) were added into the reaction system and incubated at 37 °C for 2 h. The lipids were extracted in chloroform: methanol (2:1) dried under N_2_ gas and separated by TLC using chloroform:MeOH:NH4OH (14:6:1) at room temperature for 10 min. The chromatography film was scanned after 10 min with an autoradiography system (Chemiluminescence Imaging System, Clinx Science Instruments Co., Ltd., Shanghai, China), and the intensity of each band was measured using Image-Pro Plus version 6.0 software (Media Cybernetics, Inc., Rockville, MD, USA).

### 4.4. SM Measurement

The sperm were homogenized with PBS. After being centrifuged, the supernatant was collected and used to determine the protein concentration (Wanleibio, Co., Ltd. Shenyang, China). An equal volume mixture of chloroform/methanol (2:1, *v*/*v*) was added to the supernatant to extract the total lipids. Thereafter, the supernatant was collected and then dried by nitrogen gas after being centrifuged at 8000 rpm/min, at 4, for 10 min. Finally, the content of SM was measured as previously described [41,42].

### 4.5. Sperm Motility and Penetration Tests

The normal semen samples were treated with the anti-SMS2 antibody in a 5% CO_2_ incubator for 1 h at 37 °C, and the control groups were treated with the IgG antibody. For each group, more than 200 sperm cells were counted [43]. Then, these treated cells were used to measure the motility and penetration into methylcellulose. Briefly, a computer-assisted sperm analysis (CASA) system was used to analyze sperm motility, including the total and progressive sperm motilities. Methylcellulose solution, 1% (*w*/*v*), was used for the sperm penetration assay to mimic the viscous environment of the female reproductive tract. First, the methylcellulose was introduced into 7.5 cm flattened capillary tubes (1.0-mm inner depth; Elite Medical Co., Ltd., Nanjing, China), with one end sealed with plasticine. Next, the open ends of the capillary tubes were inserted into the samples and incubated for 1 h at 37 °C. Then, the tubes were imaged with a Leica DM2500 upright microscope. Two fields, at 1 and 2 cm from the base of the tube, were recorded. A minimum of 200 sperm cells were counted for each assay.

### 4.6. Evaluation of Capacitation and the Acrosome Reaction

Sperm cells were treated with anti-SMS2 antibody as above, and 20 µM progesterone were added to stimulate their hyperactivity. Chlortetracycline (CTC) staining was used to assess the capacitation and acrosome reaction as previously described [44]. The stained cells were measured with a fluorescence emission via a DM 400 dichromatic mirror (Leica “A” filter, Heidelberg, Baden Wuertenberg, Germany) and Leica DM2500 upright microscope (Heidelberg, Baden Wuertenberg, Germany) using an Hg excitation beam including a 340–380 nm filter. In this experiment, 200 spermatozoa were calculated to estimate the different CTC staining patterns as follows: “F” corresponds to the uncapacitated cells; “B” corresponds to the characteristics of capacitated but acrosome-intact cells; and “AR” (for acrosome reaction) represents sperm cells that suffered acrosomal exocytosis. Moreover, the capacitated sperm cells were the sum of “F” and “B”.

### 4.7. Single Sperm [Ca^2+^]i Imaging

The anti-SMS2-treated and IgG-treated single sperm [Ca^2+^]i imaging was performed as previously described [45]. Briefly, when the spermatozoa swam out of the cauda epididymal, they were kept in HS solution (135 mM NaCl, 5 mM KCl, 1 mM MgSO_4_, 2 mM CaCl2, 20 mM HEPES, 5 mM glucose, 10 mM lactic acid, and 1 mM Na-pyruvate at pH 7.4 with NaOH) and were stained with 5 μM Fluo-4 AM (Molecular Probes, Eugen, OR, USA) and 0.05% pluronic F-127 (Molecular Probes, Eugen, OR, USA) for 30 min at room temperature. Subsequently, the cells were washed with HS medium containing 2 mM EGTA, but not Ca^2+^, or with HS medium. The washed cells were loaded on Cell-Tak (BD Biosciences, Lake Franklin, NJ, USA) and were allowed to attach for 20 min. A monochromator (Polychrome V, TILL Photonics GmbH, Munich, Bavaria, Germany) was used to form an excitation at 488 nm for Fluo-4 to image the sperm (IX-71, Olympus, Shinjuku, Tokyo, Japan). Emissions (515–565 nm) were collected with a cooled CCD camera (CoolSNAP HQ, Roper Scientific, Acton, MA, USA) that recorded 100 ms every 2 s. For image analysis, a commercial software was used (MetaFluor v7, Molecular Devices, San Jose, CA, USA).

### 4.8. Quantitative PCR Analysis

The total RNA of the sperm was isolated by using the RNAiso Plus reagent (Takara Biotechnology Co., Ltd., Dalian, China); about 2 μg RNA was reverse transcribed into cDNA by using a cDNA Synthesis kit (cat. no. RR047A, Takara Biotechnology Co., Ltd., Dalian, China). Primers for GAPDH, and SMS2 were synthesized by Shenggong Biology Engineering Technology Service, Ltd. (Shanghai, China), and the primer sequences were as follows: SMS2 forward TGGCAAGATGCTGTGGGATA, reverse CCAAGATCAACATGGACTCTTACA; GAPDH forward GTCGGAGTCAACGGATT, reverse AAGCTTCCCGT TCTCAG. In order to measure the expression of SMS2, SYBR green was used as a fluorescent dye (cat. no. RR42LR; Takara Biotechnology Co., Ltd.). The PCR program (ABI 7500, Thermo Fisher Scientific, Inc.) comprised of 95 °C for 3 min, and 40 cycles of 95 °C for 10 s and 60 °C for 50 s. Using the comparative quantification cycle (Cq) method, the relative expression levels were calculated using the formula for 2^−ΔΔCt^. Experimental data represent the average and standard deviation of three biological replicates. The SMS2 expression level was normalized to the expression of GAPDH [46].

### 4.9. Western Blot Analysis

The anti-SMS2-treated semen samples and the normozoospermia and asthenospermia semen samples were used to measure their protein content [45]. Briefly, the human sperm cells were washed three times with PBS, proteins were extracted using the RIPA buffer, and their concentration was measured using the BCA assay (Kangwei, Beijing, China). Protein samples were separated by sodium dodecyl sulfate-polyacrylamide gel electrophoresis (SDS-PAGE), and transferred onto polyvinylidene fluoride membranes (Immobilon P, Millipore, Boston, MA, USA). Subsequently, the membranes were blocked with 10% skimmed milk and then incubated with primary antibodies overnight at 4 °C. The following antibodies were used at the indicated dilutions: SMS2 (Abgent, Suzhou, China) at 1:1000, ERK (affbiotech, Changzhou, Jiangsu, China) at 1:800, p-ERK (affbiotech, Changzhou, China) at 1:500, Akt at 1:300 (Proteintech, Wuhan, China), p-Akt at 1:500 (affbiotech, Changzhou, Jiangsu, China), cleaved-caspase 3 at 1:500 (Proteintech, Wuhan, China), and GAPDH at 1:10,000 (Proteintech, Wuhan, China). Secondary horseradish peroxidase-coupled antibodies (rabbit, Proteintech, Wuhan, China) were used at 1:10,000. Signals were revealed using an enhanced chemiluminescence reagent (Kangwei, Beijing, China) and an autoradiography system (CLINX, Chemiluminescence Imaging System, Shanghai, China).

### 4.10. Statistical Analysis

All results were analyzed using GraphPad Prism 6.0. The differences between each group were presented by one-way analysis of variance (ANOVA). Statistical analysis was performed using an unpaired t-test. Significant differences were considered at *p* < 0.05.

## 5. Conclusions

Taken together, our study supports the role of SMS2 in regulating the sperm motility and penetration ability into methylcellulose, and participating in the Akt and ERK pathway-related apoptosis, which contributes to the development of asthenospermia.
